# On the Trail of Stubborn Bacterial Yellowing Diseases

**DOI:** 10.3390/microorganisms13102296

**Published:** 2025-10-03

**Authors:** Moshe Bar-Joseph

**Affiliations:** The S. Tolkowsky Laboratory, Department of Plant Pathology and Weed Research, Agricultural Research Organization—The Volcani Center, P.O. Box 15159, Rishon Lezion 7528809, Israel; mbjoseph@gmail.com

**Keywords:** mollicutes, citrus, papaya, carrot, ELISA, antibiotic, heat therapy, vector exclusion Koch’s postulates

## Abstract

This retrospective review traces personal encounters along the complex path of plant yellowing diseases—graft-transmissible disorders historically attributed to elusive viruses, but later linked to phloem-invading, wall-less bacteria known as Mollicutes. These include two plant-infecting genera: the cultivable *Spiroplasma* and the non-cultivable ‘Candidatus Phytoplasma’. A third group—the walled, psyllid-transmitted *Candidatus Liberibacter*—was later implicated in closely similar syndromes. This shift in understanding marked a major turning point in plant pathology, offering new insights into yellowing diseases characterized by stunting, decline, and poor or deformed growth. The review focuses on key syndromes: citrus little leaf disease (LLD), or citrus stubborn disease (CSD), caused by *Spiroplasma citri*; and several Mollicute -related disorders, including safflower phyllody, Bermuda grass yellowing, and papaya dieback (PDD) (Nivun Haamir), the latter linked to ‘Candidatus Phytoplasma australiense’. Despite differing causes and vectors, citrus LLD-CSD and PPD share an erratic, unpredictable pattern of natural outbreaks—sometimes a decade apart—hindering grower engagement and sustained control efforts. While scientific understanding has deepened, practical management remains limited. The recent global spread of Huanglongbing (HLB), caused by *Candidatus Liberibacter* species, underscores the urgent need for improved strategies to manage this resilient group of phloem-limited bacterial pathogens.

## 1. Introduction

Mollicutes were first identified as a new class of plant pathogens in Japan nearly six decades ago [1,2]. This seminal discovery reshaped the field of plant virology by introducing a novel group of insect-transmitted bacterial pathogens [3,4,5,6,7,8], restricted to the phloem tissue [8,9] of an increasing number of plants exhibiting yellowing symptoms [9]. Only a few of the previously described yellowing diseases, such as sugar beet yellows, result from viral infection [10].

Subsequent research uncovered a wide array of mollicute-associated plant diseases, including the cultivable *Spiroplasma* species [11,12,13,14,15,16,17,18], as well as a much larger group of currently uncultivable phytoplasmas [19,20,21,22,23,24,25,26]. A recent phylogenomic analysis proposed an emendation of the order *Mycoplasmatales*, into two families of *Spiroplasmataceae* and the *Mycoplasmataceae*, comprising six genera [4].

These two classes of wall-less bacteria, along with a third group—the walled, psyllid-transmitted *Candidatus Liberibacter* species—are all phloem-invading, insect-transmitted pathogens associated with closely similar yellowing and decline symptoms in plants [8,9,27,28,29,30,31,32].

The recognition of multiple, distinct bacterial agents responsible for yellowing symptoms in diverse cultivated and wild plant species has posed considerable taxonomic and microbiological challenges, and has drawn significant scientific attention.

This review summarizes the author’s personal encounters as a veteran citrus and subtropical fruit tree pathologist, with mollicutes and related pathogens. It therefore includes numerous self-citations that trace the trajectory of my past work. These references are not meant to imply particular scientific weight; indeed, many foundational and recent contributions that have significantly advanced the study of phloem-limited bacterial pathogens are only briefly referenced here.

## 2. Citrus Little Leaf (LLD)—Citrus Stubborn Disease (CSD) in Israel

Between the late 1920s and World War II, the Mediterranean citrus industry experienced significant expansion. In the coastal regions of the country, shortly after groves were planted, many young citrus trees exhibited stunting and bore misshapen fruit—a condition that was named Alelet in Hebrew [33], meaning “little leaf disease” (LLD). The LLD was later recognized as synonymous with citrus stubborn disease (CSD), which had been noticed about two decades earlier in California [34].

Initially, LLD was thought to be a physiological disorder caused by xeromorphic stress resulting from intense heat waves (hamsins), which were believed to inflict permanent damage on young citrus orchards [33]. In retrospect, this early report provided an excellent phenological description of the disease, albeit with a misinterpretation of its etiology. Later studies in California [35,36] and Israel [37,38] suggested that elevated temperatures accelerated the reproduction and population growth of mollicute-transmitting leafhoppers. This likely contributed to the migration of *Spiroplasma citri*-harboring vectors from their natural habitats toward newly planted citrus groves.

Stubborn disease was reported across the Mediterranean Basin and California [39,40,41,42,43,44,45,46]. A similar disease described in Egypt was named Safargalli (SD) [47]. From a horticultural perspective, it is noteworthy that new cases of LLD and stubborn infections were typically limited to 2- to 4-year-old trees. For this reason, the LLD problem reappeared in Israel only in the mid-1950s, when citrus planting resumed. Disease incidence varied depending on planting year and cultivar [48]. Washington Navel oranges were the most susceptible, while Shamouti oranges showed lower infection rates. Interestingly, no infections were observed in lemon groves.

As a Ph. D. student working on Citrus tristeza virus (CTV) in the late 1960s [49], one of my tasks involved indexing citrus propagation material. Inspired by a publication describing a novel detection method for SD [47], I attempted to use the technique for LLD detection—with disappointing results. This modest project became the subject of my first attempted publication: a short note submitted to Phytopathology, which was ultimately rejected. The main criticism concerned the lack of convincing evidence that the asymptomatic test trees were indeed disease-free. The experience left a lasting impression on my research career, highlighting the reality that disproving a failed hypothesis can be far more difficult than advancing a new one.

Shortly thereafter, I encountered the abstract by Doi et al. [1], which proposed a link between mycoplasma-like organisms (MLOs) and various yellowing plant diseases. I discussed the potential involvement of MLOs in LLD with my supervisor, Prof. Loebenstein, head of our laboratory. He assigned Aaron Zelcer—who had just completed his M.Sc. and developed considerable expertise in electron microscopy—to investigate the presence of MLOs in LLD-infected citrus.

Zelcer began by sectioning *Catharanthus roseus* leaves infected with safflower phyllody (SPD) [50]. He immediately observed numerous pleomorphic bodies densely packed within the *Vinca* phloem cells. Encouraged by these results, he proceeded to examine symptomatic leaves from LLD-infected Valencia oranges. However, all initial attempts to detect MLOs in symptomatic citrus tissues failed. Nearly two years later, Dr. E.C. Calavan suggested examining young, asymptomatic leaves for microscopy. This guidance proved pivotal, as reported in our subsequent publication [51].

In retrospect, one of the major challenges in detecting in LLD infected plants was the low number of *S. citri* cells in symptomatic citrus leaves, especially when compared to the abundance of the SPD causing mollicutes, in *Vinca* phloem cells [52]—a phenomenon we later documented using ELISA [53]. The underlying causes of this variability [51,52,53] remain unresolved and may hold clues for understanding and managing mollicute-related diseases.

## 3. Bermuda Grass Yellowing Disease (1970–1975)

Bermuda grass (*Cynodon dactylon*) (Bg)—a persistent weed problem in young citrus groves—was observed exhibiting yellowing symptoms, with some phloem cells heavily colonized by MLOs [54]. Attempts to propagate diseased stolons consistently failed, as the infected tissues typically died shortly after planting. In contrast, healthy Bg plants propagated easily from stolons.

Our goal was to establish a greenhouse culture of the diseased plants. We found that dipping infected Bg stolons in antibiotic solutions for several hours prior to planting [54] enabled them to survive, root, and exhibit temporary remission of symptoms. This method proved to be a simple and effective way to maintain the yellowing affected plants. The stolon-dipping technique also served as a valuable tool for qualitatively and quantitatively evaluating antibiotic treatments and for assessing the duration of symptom remission following treatment [54].

This method remained largely overlooked until decades later, when a similar approach was used to assess antibiotic effects in citrus plants infected with *Candidatus Liberibacter asiaticus* (CLas), the causal agent of citrus Huanglongbing (HLB) [55].

Another little-noticed study we conducted involved examining morphological changes in phytoplasma cells within heat-treated *Catharanthus roseus* plants infected with SPD [52]. Unlike normal phytoplasma cells, which contain visible cytoplasmic content, the pathogen cells from heat-treated plants displayed only membrane contours—completely devoid of internal contents. Although we did not pursue this line of inquiry further, it offered a simple model for studying phytoplasma membranes composition and stability—a subject that would attract considerable interest in later years [56].

## 4. *Spiroplasma citri* and the Causal Agent of LLD-CSD (1973–1974)

During my postdoctoral stay at the John Innes Institute (JII) in Norwich, UK, in 1973, my research focused on the characterization of beet yellows virus (BYV) [10,49]. At the same time, the bacteriologists at JII were attempting to culture MLOs from yellowing-infected plants. Despite multiple efforts, the only organism successfully isolated was *Acholeplasma laidlawii*, a free-living, non-pathogenic mollicute. Consequently, they expected a culture of *S. citri* [12] but had not received it.

Since a simple culture protocol for *S. citri* had already been published [57], I suggested attempting isolating the *S. citri* cells, from LLD fruits. Permission was granted to import diseased citrus fruits, which were kindly sent me by Dr. Benny Raccah. Sterile seed coats from these Valencia oranges were used to culture *S. citri* according to [57] and the fresh culture was injected into *Euscelidius mundus* leafhoppers, caged on an leguminous plants.

The caged plants eventually became stunted, producing multiple miniature leaves. Repeated culturing and electron microscopy confirmed the presence of *S. citri* cells in the dwarfed legume plants [58]. To complete Koch’s postulates, seedy oranges from the Norwich market were used to extract seeds which were propagated and the one- to two-month-old sweet orange seedlings, were caged with *S. citri*-injected leafhoppers. Within four to eight weeks, the young citrus leaves developed mottling symptoms characteristic of LLD/CSD. Electron microscopy and culture re-isolation confirmed the presence of *S. citri*, thus completing Koch’s postulates for the disease [59]. Interestingly, later studies in California showed that successful transmission by leafhopper injection was only possible with fresh *S. citri* cultures [60]. Had the Norwich lab received and used the requested aged, high-passage isolates—devoid of the transmission factor—the experiment would have failed, and Koch’s postulates for LLD/CSD would not have been fulfilled at that time. In retrospect, this was the first successful fulfillment of Koch’s postulates not only for *S. citri* but for any mollicute. The JII group shifted research interests in later years, and their important contributions to *S. citri* pathogenicity studies were mentioned and recognized less than deserved.

## 5. *Spiroplasma citri* Diagnosis Using ELISA (1978)

In 1978, we were introduced to ELISA and used it for large-scale CTV surveys [49]. Given ELISA have proven value for CTV detection; we tested the possible application of the method for compounding *S. citri* surveys within the ongoing CTV suppression program [49]. Rabbits were immunized with cultured cells of *S. citri*, and the resulting antibodies were tested in ELISA assays. Simultaneously, Dr. Michael Clark’s group conducted a similar study, leading to a collaborative paper [53]. Our calculations, based on the ELISA reaction of the resulting antibodies with cultured *S. citri* cells, indicated that about 10^5^ cells were required to generate a positive ELISA signal. Unfortunately, leaf and bark extracts from symptomatic LLD-infected citrus trees apparently contained too few *S. citri* cells for reliable ELISA detection. We shifted our attention from LLD-infected leaves to aborted seeds from *S. citri*-infected fruits. Their seed coats, after careful washing, reacted strongly in ELISA. Individual seed coats were yielding absorbance readings of around 2.0 OD at 405 nm [53].

However, testing seed coats from already symptomatic fruit had limited practical diagnostic value. We also obtained moderate ELISA reactions from *Sisymbrium irio* (London rocket), naturally infected plants, but only from those showing visibly detectable LLD symptoms [53].

Notably, we frequently observed high ELISA titers—reaching up to 3.0 OD at 405 nm—from single specimens of naturally trapped leafhoppers (kindly supplied by Dr. B. Raccah), as well as in *S. citri*-injected samples from JII [61]. However since, both the known LLD vectors and several non-vectoring leafhopper species harbored high titers, of ELISA detectable *S. citri*, the utility of ELISA in pinpointing of specific vectors responsible for local LLD spread, was limited [61]. Electron microscopy (EM) observations of *S. citri* cells in citrus and other host plants revealed its characteristic helical morphology, though typically in low numbers. In contrast, EM sections of infected leafhopper tissues often showed abundant spherical bodies that rarely resembled typical *Spiroplasma* forms in plant cells [57,58]

## 6. Natural Remission of LLD/CSD Symptoms: Is It Worth Waiting?

Our work on LLD occasionally overlapped with our studies on citrus tree dwarfing using viroids [62]. In 1984, we established a trial to evaluate the dwarfing of the citrus variety Oroblanco, using 12 replicates of three trees per treatment. Less than two years later, several of these trees showed typical symptoms of natural infection by LLD. This posed a dilemma: should we remove the infected trees, as was standard practice in commercial orchards, or retain them to preserve the integrity of the high-density trial (1000 trees per Hectare)? We chose to retain the infected trees while excluding their performance results, from the final analysis.

Nearly a decade later, during a planned thinning operation, we were unable to recognize the trees previously marked as LLD-infected. Surprisingly, almost all Oroblanco trees—manifesting the typical LLD symptoms eight years earlier—showed recovery and bore normal fruits.

## 7. Attempts to Rescue LLD-Infected Trees by Pruning and Hormonal Treatments

The symptom expression of naturally LLD infected young citrus trees is often uneven, with some branches on part of the trees, remaining symptom-free. Although complete removal of symptomatic trees was the recommended practice, we explored whether selective pruning could improve tree performance. In a trial at Bet Dagan, we pruned symptomatic branches from 5- to 7-year-old Valencia trees. However, symptoms quickly reappeared near the pruning sites. To prevent regrowth, we applied tree-hold solution of 200 ppm, naphthalene acetic acid (NAA), which effectively suppressed shoot development for the summer. By the following spring, however, symptoms emerged in previously unaffected parts of the trees. This suggested that pruning only temporarily delayed disease progression and may have inadvertently promoted systemic spread of the pathogen. Interestingly common horticultural practices such as topping and hedging have not increased LLD incidence, suggesting (I) the absence of hidden *S. citri* reservoirs in older trees and (II) vector preference to the Juvenile citrus tree stage, rather than flushes of young twigs on older citrus trees.

## 8. Failure to Cure LLD Infected Trees with Antibiotics (1971–1972)

In the mid-1970s, a grower consulted us about a few LLD-infected trees in his newly planted grove. At the time, I optimistically recommended removal but reassured him that effective antibiotic treatments were likely forthcoming, given *S. citri*’s known sensitivity to antibiotics in culture [63]. Together with Dr. B. Raccah, we conducted extensive trials, including foliar sprays with DMSO-adjuvanted antibiotic formulations to enhance uptake, as well as trunk and branch injections [64]. Although systemic distribution of the antibiotics was evident—demonstrated by phytotoxic effects in apical shoots of the injected trees—none of the treatments led to disease remission.

## 9. Experimenting with Shade Cloth Netting to Prevent LLD Infection

In a citrus dwarfing experiment at the Hula Valley Research Station, we tested netting as a preventive measure against LLD infections. Newly planted ‘Star Ruby’ grapefruit trees were either individually covered with 17% white shade nets or treated with 6–8 applications of 10% kaolin-based whitewash spray [65].

The incidence of LLD remained low (1–2%) across all treatments—including the netted, sprayed, and untreated control trees—suggesting that neither netting nor spraying significantly altered infection rates under conditions of very low natural disease pressure. One plausible explanation is that under low transmission conditions, initial infections did not occur from the airborne landing of leafhoppers directly onto the young citrus plants. Instead, infective leafhoppers may have first landed in the open spaces between the sparsely planted trees and then moved across the ground to adjacent plants, not sufficiently protected to prevent infections starting from the ground face.

This hypothesis aligns with accumulated experience indicating that new LLD symptom observations are largely restricted to the 2–4 years post-planting period, when tree spacing is wide and inter-row ground remains exposed. In contrast, older, denser groves with closed canopies rarely show new infections.

## 10. Clustering of LLD-Infected Trees

Between 1994 and 1995, some of the newly planted citrus groves in the Northern Negev exhibited LLD infection rates of up to 50%. The spatial distribution of the LLD trees [66] revealed a strong tendency for infected trees to appear in small clusters.

Since *S. citri* is not naturally transmitted by the leafhopper vectors from the LLD-infected trees, we proposed an alternative mechanism for the LLD clustering: the migrating leafhoppers carrying *S. citri* initially land and transmit the pathogen to the accessed widely spaced young trees. Since the vector finds the invaded citrus trees unsuitable for reproduction, they leave the initially infected trees and move to continue probing of nearby trees, rendering them sequentially infected. This model could explain why, even in seasons with low LLD infection rates of 1–2%, a significant proportion of the few affected trees appear frequently in close proximity.

## 11. Papaya Dieback-Nivun Haamir (1982–1991)

In the summer of 1982, papaya cultivar trials were conducted at the Gilgal station in the Jordan Valley. By October, Drs Reuveni and Lavie observed, widespread decline across multiple papaya genotypes. Affected plants exhibited progressive yellowing, fruit drop, and eventual death, resembling papaya dieback disease (PDD) a previously reported malady in Australia [67]. Initial symptoms included slight apical curvature, followed by rapid yellowing of the upper foliage. Within four weeks, apical necrosis, fruit browning, and complete yield loss occurred.

Although based on the Australian experience the malady was suspected to be associated with a soil-borne cause [67]. Initial field observations did not support this. The patchy distribution of affected plants was inconsistent with typical soil-related problems. Moreover, we observed that the few papaya plants with two to three branches (due to earlier mechanical stem damages) showed dieback of only one branch, while the others remained healthy—suggesting an airborne, rather than soil-borne, agent. The restricted seasonal window for infection—four to six weeks starting in late October, with a secondary, milder peak in May—further suggested a vector-borne pathogen. Electron microscopy of symptomatic tissues revealed structures resembling rhabdovirus particles, but these could not be reliably associated with disease. Mechanical inoculation of herbaceous indicator plants and dsRNA analysis failed to associate the problem with recorded virus infections. Grafting experiments produced only one successful graft take and transmission, rendering disease etiology inconclusive [65]. In 1983, we conducted a six-treatment insect exclusion experiment at the Gilgal station to test the vector hypothesis:

1. Insect-proof netting
2. 30% shade netting
3. 15% shade netting
4. 15% shade netting + Temik (aldicarb) soil insecticide
5. Whitewash spraying every 10 days
6. Uncovered controls

The results were definitive: while the uncovered control plot exhibited ~40% infection, all protected treatments had infection rates of only 1–2% [65]. This strongly supported a vector-mediated transmission pathway, despite the causal agent remaining unidentified. Whitewash spraying—originally developed to deter aphid landings was also effective in reducing infection. However, the need for frequent re-application and the buildup of kaolin layers increased susceptibility to mite infestations.

Following these findings, commercial papaya plantations in the Jordan Valley adopted coarse (15%) netting, which effectively controlled PDD. However, two major drawbacks emerged:

1. Dust accumulation on nets significantly reduced light transmission, resulting in plant etiolation.
2. Netting interfered with insect pollination, leading to poor-size fruit set.

By 1987, just three years after introducing the coarse net protection practice, natural PDD spread had declined significantly, and annual incidence of the disease in the non-covered papaya plots was negligible, rendering the continued use of netting unnecessary.

The effectiveness of physical exclusion measures pointed to a mollicute etiology. However, several electron microscopy attempts revealed Mollicute structures in only one symptomatic papaya sample [65], Hence their inconsistent presence precluded definitive diagnosis. Only later, with the development of PCR-based assays, was the presence of phytoplasma sequences consistently confirmed, first in Australian [68] and later in Israeli [69] samples.

## 12. Carrot Yellowing and Vector Exclusion Studies

Carrot yellowing diseases were historically attributed to phytoplasmas, and later to *Spiroplasma*, both mollicutes transmitted by leafhoppers [69,70,71].

**Figure 1:**
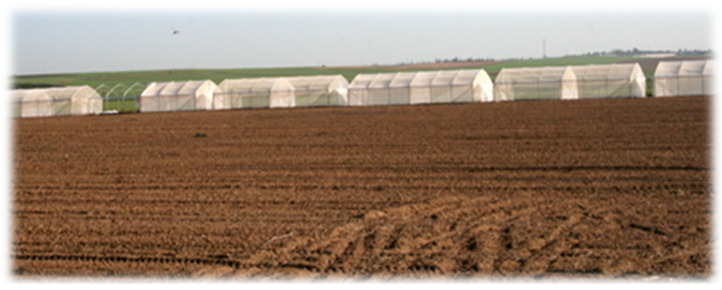
The experimental set up of two different netting systems in Kibbutz Sa’ad 2012, used to monitor the incidence and timing of C. *Liberibacter solanacearum* caused carrot yellows infections by the prevalent psyllid vector *Bactericera trigonica*. Special thanks to Dr Eli Shlevin and Erez Ben Noon, Kibbutz Sa’ad, and the Field Crop Growers Association and Chief Scientist office, Ministry of Agriculture for supporting this project.

Infections by the psyllid- transmitted carrot yellowing bacterium *‘Candidatus Liberibacter solanacearum* of carrots was suspected in Israel since 2009 (Mawassi and Bar-Joseph, unpublished). In 2012, a suppression project of *Candidatus Liberibacter solanacearum* associated infections was initiated, in which we tested the use of different netting systems to monitor the incidence and timing of the disease spread. Metal-framed enclosures (approximately 60 m^2^ each), covered with either 50-mesh insect-proof netting or 17% shade cloth, were installed near commercial carrot fields at Kibbutz Sa’ad (Figure 1). Psyllids, the vectors of *Candidatus Liberibacter solanacearum*, were able to penetrate the 17% shade cloth netting, at about 50% reduced rate compared to the uncovered control. However, the psyllids were completely excluded by the 50-mesh screen.

**Figure 2:**
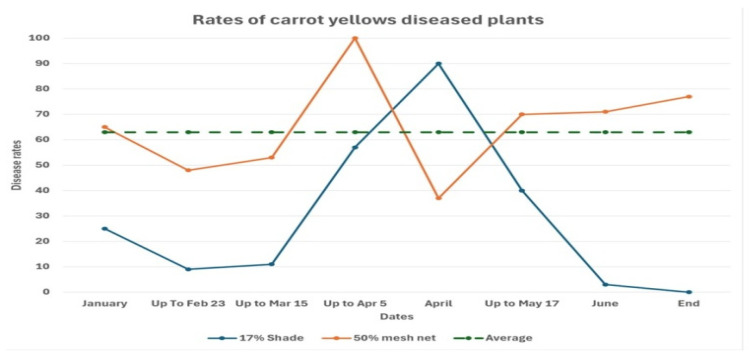
The rates of carrot yellows diseased plants in the serially exposed enclosures covered by the 17% and 50 meshes white nets. *Y*-axis: The weighted infection rates in the different plots, where a massive infection of a given plant was assigned a value of 3, and a mild infection was assigned a value of 1. *X*-axis: The time points at which the various plots were exposed, each for a period of about three weeks. The infection rate in the control plot which was uncovered over the entire of the experiment was 220.

The trial confirmed that *Liberibacter* transmission was effectively blocked by the 50-mesh net, underscoring the utility of physical exclusion against psyllid-borne pathogens. To further explore the seasonality of disease pressure, the insect proof screen-protected enclosures were deliberately serially exposed to vector entry for approximately three weeks during the growing season. This temporal exposure revealed that the primary period of carrot yellowing spread in the Sa’ad region occurred between 15 March and 15 May (Figure 2). A follow-up experiment in 2013 confirmed these findings. Moreover, it demonstrated that timely insecticide applications during peak transmission periods successfully mitigated the psyllid damage in the temporarily exposed plots [72].

These results clearly demonstrated that the value of netting extends beyond the continuous exclusion of insect vectors. As shown in the PDD and carrot yellowing trials, temporary or seasonal netting can also function as a diagnostic tool, helping to suggest an airborne pathogen and pinpoint the timing of pathogen ingress. This approach could be adapted to other airborne vector-borne diseases, providing critical insights for optimizing the timing of protective interventions such as targeted insecticide applications. Rather than relying solely on year-round physical barriers, integrating netting into both surveillance and management protocols could significantly strengthen disease control strategies.

## 13. Challenges Ahead in Translating Bacterial Genomic Advances into Practical Tools Against Phloem-Invasive Pathogens

Recent reviews [16,21,22,25,26] have highlighted major advances in the genomics of phloem-invading bacteria, including improved diagnostic techniques, the discovery of phytoplasma effectors, and new insights into the molecular mechanisms by which these pathogens manipulate their hosts. Yet, for growers confronted with the devastating reality of phloem-invading bacterial diseases, these considerable advances need still to be raised into means of practical disease relief. The remaining gap between laboratory progress and practical solutions was demonstrated in Florida and Brazil, where millions of citrus trees have been lost to huanglongbing (HLB), spread by the Asian citrus psyllid (*Diaphorina citri*) transmitting ‘*Candidatus Liberibacter asiaticus*’.

The two mollicute pathogens central to this review—*S. citri*, the causal agent of LLD-CSD, and the phytoplasma agent of PDD—differ markedly in host range, transmission biology, and pathology. Both diseases, however, are transmitted by leafhoppers that do not persist or reproduce on their cultivated hosts. As a result, infection incidence varies dramatically from year to year and is strongly influenced by vector movement from surrounding vegetation. The decadal peaks in LLD-CSD and PDD resemble large-scale insect migration events, suggesting that climatic or ecological factors may synchronize vector dispersal from wild vegetation into crop systems. Testing this hypothesis will require studies on insect neurobiology to identify markers of pre-migration triggers.

In LLD, and PDD infection rates fluctuate widely—ranging from 10 to 50% during severe outbreaks to negligible levels in other years. In LLD the new infections are largely confined to young trees (aged 2–4 years). Symptom remission also varies by cultivar: ‘Oroblanco’ sometimes exhibits near-complete remission, while the ‘Valencia’ trees remain mostly symptomatic for extended periods. This inconsistency likely reflects incomplete systemic colonization by *S. citri*. Notably, recovery of apple trees from apple proliferation phytoplasma has been linked to increased callose deposition, which prevents recolonization of tree crowns during spring [73].

Despite such host–pathogen complexities, a striking commonality emerges: taxonomically diverse bacterial pathogens—including *S. citri* [43,44,50,52], the PDD phytoplasma agent [9,58], and ‘*Candidatus Liberibacter asiaticus*’ [27]—are typically present at very low titers, even in plants with severe symptoms. By contrast, the SPD phytoplasma and the Bg yellowing agent [52,54] accumulate at high levels within phloem cells. This discrepancy suggests that in partially resistant to mollicute abundance hosts, disease expression may be triggered by localized hypersensitive responses to low pathogen titers, with severity reflecting the host’s immune response rather than absolute pathogen load. Modern transcriptional profiling could help resolve this question by comparing gene expression patterns in hosts sustaining high versus low mollicute titers—an area that remains largely unexplored but holds significant promise.

## 14. Epilog

This article reflects on nearly sixty years of work in plant pathology. It focuses on the period when the yellowing diseases were first established as bacterial in origin, with research at that time largely conducted by scientists trained in phytopathology. While this paper has emphasized my own encounters with mollicute-associated diseases, it also seeks to indicate the challenges ahead in translating genomics, molecular biology, and vector ecology discoveries into effective field solutions for the taming of phloem-invading bacterial pathogens.
